# Chewing lice of genus *Ricinus* (Phthiraptera, Ricinidae) deposited at the Zoological Institute of the Russian Academy of Sciences, Saint Petersburg, Russia, with description of a new species

**DOI:** 10.1051/parasite/2016007

**Published:** 2016-02-22

**Authors:** Miroslav Valan, Oldrich Sychra, Ivan Literak

**Affiliations:** 1 Department of Biology and Wildlife Diseases, Faculty of Veterinary Hygiene and Ecology, University of Veterinary and Pharmaceutical Sciences Palackeho tr. 1/3 612 42 Brno Czech Republic

**Keywords:** *Ricinus vaderi*, New species, *Melanocorypha calandra*, Phthiraptera, Chewing lice, Blagoveshtchensky’s collection

## Abstract

We revised a collection of chewing lice deposited at the Zoological Institute of the Russian Academy of Sciences, Saint Petersburg, Russia. We studied 60 slides with 107 specimens of 10 species of the genus *Ricinus* (De Geer, 1778). The collection includes lectotype specimens of *Ricinus ivanovi* Blagoveshtchensky, 1951 and of *Ricinus tugarinovi* Blagoveshtchensky, 1951. We registered *Ricinus elongatu*s Olfers, 1816 *ex Turdus ruficollis*, *R. ivanovi ex Leucosticte tephrocotis* and *Ricinus serratus* (Durrant, 1906) *ex Calandrella acutirostris* and *Calandrella cheleensis* which were not included in Price’s world checklist. New records for Russia are *R. elongatus ex Turdus ruficollis*; *Ricinus fringillae* De Geer, 1778 *ex Emberiza aureola*, *Emberiza leucocephalos*, *Emberiza rustica*, *Passer montanus* and *Prunella modularis*; *Ricinus rubeculae* De Geer, 1778 *ex Erithacus rubecula* and *Luscinia svecica*; *Ricinus serratus* (Durrant, 1906) *ex Alauda arvensis.* New records for Kyrgyzstan are *R. fringillae ex E. leucocephalos* and *ex Fringilla coelebs.* A new record for Tajikistan is *R. serratus ex Calandrella acutirostris*. The new species *Ricinus vaderi* Valan n. sp. is described with Calandra lark, *Melanocorypha calandra*; from Azerbaijan, as a type host.

## Abstract (fr)

Nous avons révisé une collection de mallophages déposée à l’Institut de zoologie de l’Académie des Sciences de Russie, Saint-Pétersbourg, Russie. Nous avons examiné 60 lames avec 107 spécimens de dix espèces du genre *Ricinus* (De Geer, 1778). La collection contient des spécimens lectotypes de *Ricinus ivanovi* Blagoveshtchensky, 1951 et de *Ricinus tugarinovi* Blagoveshtchensky, 1951. Nous avons enregistré *Ricinus elongatus* Olfers, 1816 *ex Turdus ruficollis*, *R. ivanovi ex Leucosticte tephrocotis* et *Ricinus serratus* (Durrant, 1906) *ex Calandrella acutirostris* et *Calandrella cheleensis* qui avaient été omis dans la liste mondiale de Price. Les nouvelles mentions pour la Russie sont *R. elongatus ex Turdus ruficollis* ; *Ricinus fringillae* De Geer, 1778 *ex Emberiza aureola*, *Emberiza leucocephalos*, *Emberiza rustica, Passer montanus* et *Prunella modularis* ; *Ricinus rubeculae* De Geer, 1778 *ex Erithacus rubecula* et *Luscinia svecica*; *Ricinus serratus* (Durrant, 1906) *ex Alauda arvensis*. Une nouvelle mention pour le Tadjikistan est *R. serratus ex Calandrella acutirostris*. La nouvelle espèce *Ricinus vaderi* Valan n. sp. est décrite avec l’alouette calandre *Melanocorypha calandra*, d’Azerbaidjan, comme hôte-type.

## Introduction

*Ricinus* De Geer, 1778 (Phthiraptera: Amblycera) is the largest genus of chewing lice found parasitizing Passeriformes [17]. Whereas chewing lice mainly feed on feathers, these lice also feed on blood and have an unbalanced sex ratio with approximately 1 male to 10 females [17]. The distribution of many chewing lice is characterized by high host specificity and wide host distribution is therefore relatively rare [19]. Hopkins [10] noted that *Ricinus* has an anomalous distribution and occurs on approximately one-third of the 70 families of Passeriformes. Major revisions of this genus were done separately for Old World [20] and New World species [17] and comprise the majority of known species.

Price et al. [19] listed 65 species of the genus *Ricinus* parasitizing 271 different hosts in 32 families and created 299 host-louse associations. In the years thereafter, *Ricinus facetus* Mey, 2007, *Ricinus gutheili* Mey, 2007, *Ricinus nhillensis* Mey, 2007, *Ricinus ornatulus* Mey, 2007, *Ricinus ptilotulae* Mey, 2007 [16] and *Ricinus ruficapillus* Oniki, Mey, Willis, 2004 [18] were described. New host-louse associations have been reported for *Ricinus arcuatus* (Kellogg and Mann, 1912), *Ricinus diffusus* (Kellogg, 1896), *Ricinus fringillae* De Geer, 1778, *Ricinus invadens* (Kellogg, 1899), *Ricinus marginatus* (Children, 1836), *Ricinus meinertzhageni* Rheinwald, 1968, *Ricinus mugimaki* (Uchida, 1915) and *Ricinus pessimalis* Eichler, 1956, [7, 9, 11, 22–25] and these bring the total number of species to 71 and host-louse associations to 315.

Following host nomenclature according to Clements et al. [5], lice of the genus *Ricinus* infest more than one-third of the families of Passeriformes (45 of 122), still in accordance with Hopkins [10] statement. For example, *R. fringillae* De Geer, 1778 is known from 48 bird species from 9 families [11, 19]. There is no doubt that descriptions of new species and new host-louse records are expected. Consequently, examining museum collections and revision of material deposited worldwide are necessary to obtain more data concerning geographical distribution, biodiversity and host associations of chewing lice. Furthermore, for some of the species, Blagoveshtchensky reported more specimens than we have found in his collection. We hope that with our article, these lost samples will be found in the future.

Blagoveshtchensky [3] described *Ricinus ivanovi* Blagoveshtchensky, 1951 and *Ricinus tugarinovi* Blagoveshtchensky, 1951; he also mentioned additional records of this genus in the former USSR. Although he noted that *Calandrella acutirostris acutirostris* Hume, 1873 (Passeriformes: Alaudidae) was a host of *Ricinus serratus* (Durrant, 1906), this was not cited by Price et al. [19]. Price et al. [19] included only reliable sources, but because *R. serratus* is already known from several alaudid hosts, this prompted us to inspect material acquired by Blagoveshtchensky. These specimens are deposited at the Zoological Institute of the Russian Academy of Sciences, Saint Petersburg (ZISP) and are part of a larger collection, mainly assembled by him during the 1930s through the 1970s.

In this article, we present data obtained from the studies of *Ricinus* spp. deposited at the ZISP. We include new country records for Azerbaijan, Kyrgyzstan, Russia and Tajikistan. Knowing that data about the biodiversity of chewing lice within the former USSR were published mainly in Russian and that the existing literature is scarcely accessible, we will refer only to those records we have been able to verify. Accordingly, species of *Ricinus* recorded so far in Azerbaijan are *Ricinus frenatus* Burmeister, 1838 *ex Regulus regulus buturlini* and *R. fringillae ex Emberiza schoeniclus* [2]. Records in Russia are *Ricinus elongatus* (Olfers, 1816) *ex Turdus pilaris* L., 1758 and *Turdus merula* L., 1758; *R. fringillae ex Fringilla coelebs* L., 1758 and *Ricinus thoracicus* Packard, 1870 *ex Plectrophenax nivalis* (L., 1758) [4, 14]. Records from Tajikistan are *Ricinus frenatus* (Burmeister, 1838) *ex Regulus regulus* (L., 1758); *R. fringillae ex Fringilla coelebs*, *Emberiza citrinella* L., 1758, *Emberiza cia* L., 1758 and *Prunella collaris* (Scopoli, 1769); *R. ivanovi ex Leucosticte brandti* Bonaparte, 1850; *R. serratus ex C. a. acutirostris* and *Galerida cristata* (L., 1758); *R. tugarinovi ex Terpsiphone paradisi* (L., 1758) [3].

## Materials and methods

Chewing lice of the genus *Ricinus* deposited at the ZISP were examined and described. In this article, we present all *Ricinus* species found in this collection. Host systematics follow Clements et al. [5]. To each louse species, we added notes as follows: number of females, males, nymphs, host (Order: Family) including common English name, country, location, number of specimens (slide number), date, collector (coll.) and identifier (det.). Slide numbers are equal to accession numbers, but only handwritten catalogues exist. For those slides without slide numbers, we allocated a new slide number in the form MVXY. Notes from slides about locality are rewritten from slide labels while transliterating from Cyrillic to the Latin alphabet without further changes, and we cannot guarantee their validity. Where information may be lacking, this should be considered as a deficiency of information noted by the collector or collection manager. The majority of the examined specimens are in poor condition, mainly due to mounting directly to medium without using any clearing agent prior to mounting. The medium is most likely Canada balsam, but there are no written notes about it. If the case happened to be otherwise, this will be noted below.

Species concept, morphological characters and system of chaetotaxy follow Nelson [17] (see Fig. 2). All measurements are in millimeters and were taken using QuickPhoto Micro 3.0. In order to achieve high quality, drawings were made as vectors using Adobe Illustrator C6.

The newly described chewing louse species is attributed to the first author.

## Results and discussion

In total, we examined 107 specimens of the genus *Ricinus* mounted on 60 slides and no samples in fixatives were found. The majority of these specimens (78) are females, with only 7 males and 22 nymphs. Specimens had been collected from various locations and by several collectors. Specimens from Tajikistan had mostly been examined and data presented by Blagoveshtchensky [3]. Studies of lice obtained in Azerbaijan have also been published [2], with the exception of *Ricinus* sp. from *Melanocorypha calandra.* As expected, the sex ratio is unbalanced (1–11.14) and our results correspond with Nelson [17] and literature cited therein, thus showing that females of *Ricinus* are approximately 10 times more abundant than males.

Phthiraptera Haeckel, 1896

Amblycera Kellogg, 1896

Ricinidae Neuman, 1890

*Ricinus* De Geer, 1778

### *Ricinus dolichocephalus* (Scopoli, 1763)

*Pediculus dolichocephalus* Scopoli, 1763: 382.

Type host: *Oriolus oriolus* (L., 1758) – Eurasian Golden Oriole (Oriolidae).

Type locality: NE Poland.

Material examined: 1♀ (slide no. 158) *ex O. oriolus* (Passeriformes: Oriolidae), Eurasian Golden Oriole. Russia, Crimea.

Remarks: According to literature to which we had access, this is the first record of *R. dolichocephalus* in Russia.

### *Ricinus elongatus* (Olfers, 1816)

*Nirmus elongatus* Olfers, 1816: 88.

Type host: *Turdus viscivorus* (L., 1758) – Mistle Thrush (Turdidae).

Type locality: Hodonín, Czech Republic.

Material examined: 6♀, 1♂, 1N.

1♀ (slide no. MV01) *ex Turdus ruficollis* Pallas, 1776 (Passeriformes: Turdidae), Red-throated Thrush. Russia. Stanoviy khrebet: 9.V.1944.

5♀, 1♂, 1N *ex Turdus* sp. (Passeriformes: Turdidae), Russia. Okr. Turukhanska: 3♀, 1♂, 1N (slide no. MV02), 30.V.1902, Ostrovskikh P. coll.; Tomskaya gub. Elizavetinsk. zav.: 1♀ (slide no. MV03), 5.VII.1903; no specific location: 1♀ (slide no. MV04), 1907, Haritonov coll.

Remarks: Grube [8] reported *Physostomum mystax* (junior synonym of *R. elongatus*) on *T. ruficollis.* Balát [1] in his study, cited Grube [8] and included *T. ruficollis* as a host of *R. elongatus* without examining any material. In revision of *Ricinus* occurring in the Old World [20], Rheinwald did not report any notes about this association and this is probably why it was not cited by Price et al. [19]. We have unfortunately not been able to check Grube’s notes and assume that specimens are not available for study. Having previous unreliable reports and only one specimen in this collection creates an aggravating circumstance. However, *R. elongatus* is known as a parasite of 11 species of birds from four closely related families, and eight of these are from the genus *Turdus* (Turdidae). Bearing this in mind, we suggest that *T. ruficollis* be recognized as a valid host for *R. elongatus*.

### *Ricinus frenatus* (Burmeister, 1838)

*Physostomum frenatum* Burmeister, 1838: 442.

Type locality: Not recorded (probably Germany).

Type host: *Regulus regulus* (L., 1758) – Goldcrest (Regulidae).

Material examined: 10♀.

5♀, *ex R. regulus* (Passeriformes: Regulidae), Goldcrest. Tajikistan. Zap. Tigrovaya Balka: 1♀ (slide no. 134), 21.XII.1939; 1♀ (slide no. 135), 3.I.1940; 3♀ (slide no. 136), 10.I.1947, Ivanov A. coll., det. Blagoveshtchensky.

5♀ *ex Regulus r. buturlini* Loudon, 1911 (Passeriformes: Regulidae), Goldcrest (Caucasian). Azerbaijan. Lenkoranskiy r-n d. Alekseevka: 3♀, 10–14.III.1934 (slides no. 130–132), Shtrom Zh. coll., det. Blagoveshtchensky; Sari Island (Caspian Sea): 2♀ (slide no. 133), 14.III.1937, det. Blagoveshtchensky.

Remarks: Specimens were examined and notes were given by Blagoveshtchensky [2, 3].

### *Ricinus fringillae* De Geer, 1778

*Ricinus fringillae* De Geer, 1778: 71.

Type host: *Emberiza citrinella* L., 1758 – Yellowhammer (Emberizidae).

Type locality: Saxony, Germany.

Material examined: 30♀, 3♂, 14N.

1♀, 4N (slide no. 2410) *ex Carduelis flammea* (L., 1758) (Passeriformes: Fringillidae), Common Redpoll. 17.I.1929.

1♀, 7N (slide no. MV05) *ex Emberiza aureola* Pallas, 1773 (Passeriformes: Emberizidae), Yellow-breasted Bunting. Russia. Okr. n-Tambovska na Amure: 4.VIII.1939, Blagoveshtchensky coll.

2♀, 1♂ (slide No. 156) *ex E. citrinella* (Passeriformes: Emberizidae), Yellowhammer. Tajikistan. Dushanbe: 15.II.1947, Ivanov A. coll., det. Blagoveshtchensky.

1N (slide no. MV06) *ex Emberiza leucocephalos* Gmelin, 1771 (Passeriformes: Emberizidae), Pine Bunting. Kyrgyzstan. Naryn: 9.III.1913.

2♀ (slide no. MV07) *ex Emberiza rustica* Pallas, 1776 (Passeriformes: Emberizidae), Rustic Bunting. Russia. Ural Basseg: 14.VII.1948.

1♀ (slide no. 155) *ex Emberiza schoeniclus* (L., 1758) (Passeriformes: Emberizidae), Reed Bunting. Azerbaijan. Kumbashi: 28.II.1934, Shtrom Zh. coll., det. Blagoveshtchensky.

15♀, 1♂, 2N, *ex Fringilla coelebs* L., 1758 (Passeriformes: Fringillidae), Common Chaffinch.

Russia. Petrodvorec: 3♀ (slides no. MV08–10), VI.1952, Karakasheva coll; Tver’ okr.: 1N (slide no. 139), 18.IV.1907, det. Blagoveshtchensky; and 1♀, 1♂ (slide no. 137), 13.IX.1907, Plotnikov V. coll., det. Blagoveshtchensky.

Kyrgyzstan. Naryn: 1♀ (slide no. MV011), the most likely III.1913.

Tajikistan. k. Tazhni: 3♀ (slide no. 138), 25.II.1947, Ivanov A. coll., det. Blagoveshtchensky.

No locality notes: 1♀ (slide no. MV12).

Locality: 6♀, 1N (slide no. 2575) *ex F. coeleb*s (Passeriformes: Fringillidae), Common Chaffinch. Tsikhis-Dzhivari: 1935.

3♀ (slide no. MV13) *ex Passer montanus* (L., 1758) (Passeriformes: Passeridae), Eurasian Tree Sparrow. Russia. Vologda: 1947.

1♀, 1♂ (slide no. 154) *ex Prunella collaris* (Scopoli, 1769) (Passeriformes: Prunellidae), Alpine Accentor. Tajikistan. Gissarskiy khrebet pereval Anzob: VIII.1945, Ivanov A. coll., det. Blagoveshtchensky.

3♀ (slide no. MV14) *ex Prunella modularis* (L., 1758) (Passeriformes: Prunellidae), Dunnock. Russia. Kavkaz: 1917, Satunin coll.

1♀ (slide no. MV15) *ex Turdus* sp. (Passeriformes: Turdidae). Ukraine. Kiev: 3.IV.1902.

Remarks: According to literature to which we had access, new host records for *R. fringillae* in Russia are *Emberiza aureola*, *E. leucocephalos*, *E. rustica*, *P. montanus* and *P. modularis*.

### *Ricinu*s *ivanovi* Blagoveshtchensky, 1951 (Fig. 1A)

*Ricinus ivanovi* Blagoveshtchensky, 1951: 283.

**Figure 1.**
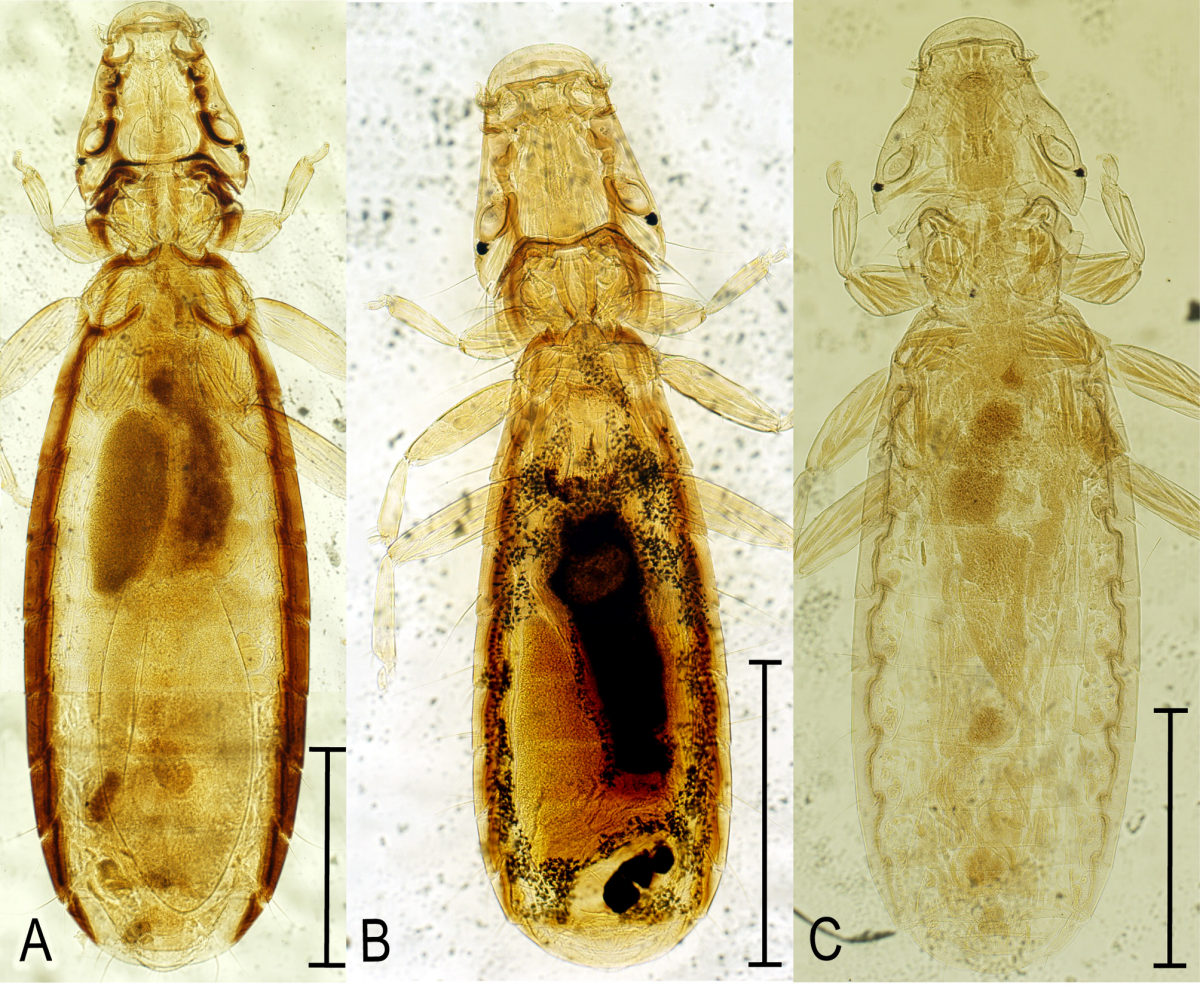
(A) Lectotype female of *Ricinus ivanovi* Blagoveshtchensky, 1951. (B) Lectotype female of *R. tugarinovi* Blagoveshtchensky, 1951. (C) Holotype female of *R. vaderi* n. sp. Scale bar: 1 mm.

Type host: *Leucosticte brandti* Bonaparte, 1851 – Black-headed Mountain-Finch (Fringillidae).

Type locality: Vewrk, Gissar’s Range, Pamir, Tajikistan.

Material examined: 1♀ (slide no. 152) *ex L. brandti* (Passeriformes: Fringillidae), Black-headed Mountain-Finch. Tajikistan. Gissarskiy khrebet pereval Anzob: 19.VIII.1945, Ivanov A. coll., det. Blagoveshtchensky.

Remarks: Blagoveshtchensky [3] based his description of *R. ivanovi* on 1♀. Unfortunately, Blagoveshtchensky did not provide a slide number. Neither Rheinwald [20] nor Nelson [17] examined the type specimen. The specimen examined in this study has the same notes about locality and date as given in the original description. Accordingly, this specimen is designated a lectotype specimen of *R. ivanovi.* Nelson [17] examined 8♀ and 3♂ of *R. ivanovi ex Leucosticte tephrocotis* (Swainson, 1832) and none from *L. brandti.* Nelson’s record was included by Emerson [6] in a chewing lice checklist of North America. Nevertheless, this was probably missed accidentally by Price et al. [19].

### *Ricinus rubeculae* (Schrank, 1776)

*Pediculus rubeculae* Schrank, 1776: 115.

Type host: *Erithacus rubecula* (L., 1758) – European Robin (Muscicapidae).

Type locality: Moravia, Czech Republic.

Material examined: 3♀.

2♀ (slides no. MV16–17) *ex E. rubecula* (Passeriformes: Muscicapidae), European Robin. Russia. Teberda: 13.VI.1917, Gorbunov G. coll.

1♀ (slide no. MV18) *ex Luscinia svecica* (L., 1758) (Passeriformes: Muscicapidae), Bluethroat. Russia. Kazalinsk: 15.VI.1932, Popov coll.

Remarks: According to literature to which we had access, this is the first record of *R. rubeculae* in Russia.

### *Ricinus serratus* (Durrant, 1906)

*Physostomum serratum* Durrant, 1906: 528.

Type host: *Eremophila alpestris* (L., 1758) – Horned or Przewalski’s Lark (Alaudidae).

Type locality: Ft. Collins, Colorado, USA.

Material examined: 18♀, 3♂, 4N.

1♀ (slide no. MV19) *ex Alauda arvensis* (L., 1758) (Passeriformes: Alaudidae), Sky Lark. Russia. Verhojanskij okrug: 2.VI.1927, Tkachenko coll.

2♀ (slide no. 151) *ex Calandrella acutirostris acutirostris* Hume, 1873 (Passeriformes: Alaudidae), Hume’s Lark. Tajikistan. Dushanbe okr.: 18.X.1946, Ivanov A. coll., det. Blagoveshtchensky.

15♀, 3♂, 4N, *ex Galerida cristata* (L., 1758) (Passeriformes: Alaudidae), Crested Lark. Tajikistan. Zap. Tigrovaya Balka: 1N (slide no. 143), 29.IV.1934, and 13♀, 2♂, 4N (slides no. 140–142, 144–148), 9.II.1941, Sosnina E. coll., det. Blagoveshtchensky; Kirovobad: 1♀, 1♂ (slide no. 150), 29.VI.1932, Shtrom Zh. coll., det. Blagoveshtchensky; Saray-Kamar: 1♀ (slide no. 149), 29.VI.1932, Pospelova M. coll., det. Blagoveshtchensky.

Remarks: Chewing lice on *C. a. acutirostris* and *G. cristata* in Tajikistan have been recorded [3]. The record of *R. serratus ex C. a. acutirostris* was not provided in the checklist by Price et al. [19]. *Ricinus serratus* is known from 14 species of birds from three families of which nine species belong to the family Alaudidae [19]. The world checklist [19] included two hosts from the genus *Calandrella* and besides *C. a. acutirostris*, the records for *Calandrella cheleensis* (Swinhoe, 1871) recorded by Mey [15] were missed. Mey had found only two nymphs [15] and that could possibly be the reason for excluding the record from the checklist. Nevertheless, even juvenile instars of *R. serratus* are easily distinguishable from other *Ricinus* species by the presence of unique serrated pleural nodi. We suggest *C. a. acutirostris* and *C. cheleensis* be considered as valid hosts of *R. serratus*.

### *Ricinus subdiffusus* Nelson, 1972

*Ricinus subdiffusus* Nelson, 1972: 97.

Type host: *Spizella passerina* (Bechstein, 1798) – Chipping Sparrow (Emberizidae).

Type locality: Hopland Field Station, Mendocino Co., California, USA.

Material examined: 1♀ (slide no. MV20) *ex Emberiza* sp. (Passeriformes: Emberizidae). Russia. Chukot. p-ov, Lavrentiya: 28.VII.1948, Ljobin coll.

Remarks: Although this species infests members of the family Emberizidae, we have found no previous records of it infesting birds of the genus *Emberiza*. Having just one specimen and doubtful collection techniques in the past, we suggest that *R. subdiffusus* not be considered as a parasite of *Emberiza* at this time.

### *Ricinus thoracicus* (Packard, 1870)

*Nirmus thoracicus* Packard, 1870: 94.

Type host: *Plectrophenax nivalis* (L., 1758) – Snow Bunting (Emberizidae).

Type locality: Not recorded, USA.

Material examined: 1♀ (slide no. 157) *ex P. nivalis* (Passeriformes: Emberizidae), Snow Bunting. Russia. o-v Vrangelya b. Rodzhersa: 7.V.1939, Portenko coll., det. Blagoveshtchensky, slide no. 157.

Remarks: This specimen was examined and mentioned by Blagoveshtchensky [4].

### *Ricinus tugarinovi* Blagoveshtchensky, 1951 (Fig. 1B)

*Ricinus tugarinovi* Blagoveshtchensky, 1951: 286.

Type host: *Terpsiphone paradisi* (L., 1758) – Asian Paradise-Flycatcher (Monarchidae).

Type locality: Kondara, Tajikistan.

Material examined: 1♀ (slide no. MV153) *ex T. paradisi* (Passeriformes: Monarchidae), Asian Paradise-Flycatcher. Tajikistan. Kondara: 6.VI.1944, Blagoveshtchensky coll., det. Blagoveshtchensky.

Remarks: Blagoveshtchensky [3] based his description of *R. tugarinovi* on 2♀, but he did not provide a slide number for the type specimen. Subsequently, Rheinwald [20], in his revision of the Old World species of *Ricinus*, re-described *R. tugarinovi* without examining the type material. Since the specimen examined in this study shares notes about locality and date with two specimens examined by Blagoveshtchensky [3], and inasmuch as we lack information on where the other specimen is deposited, the remaining specimen mounted on slide no. 153 is designated as a lectotype.

### *Ricinus vaderi* Valan n. sp. (Figs. 1C; 2A–2E)

urn:lsid:zoobank.org:act:F18FF85A-2A81-4CE1-8A47-4CE5E4816708

**Figure 2.**
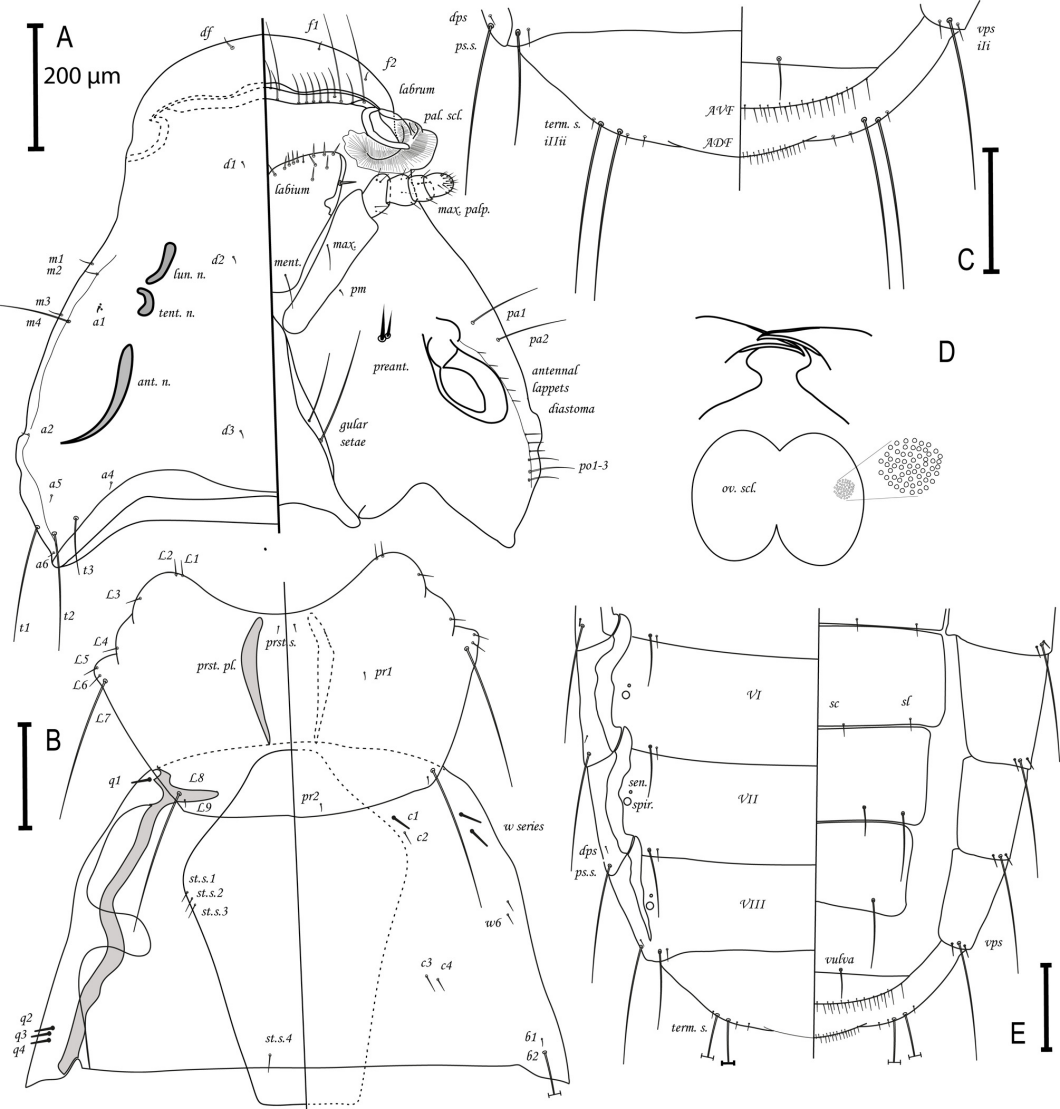
*Ricinus vaderi* n. sp. ♀ from *Melanocorypha calandra*. (A) Head, dorsoventral view: *df*, dorsal setae of the frons; *f*, ventral setae on the frons; *a*, setae on the temples dorsally; *d*, setae on the dorsum of the head; *t*, setae positioned dorsolaterally on temples; *m*, setae dorsoventrally on the marginal carinae; *po*, postocular setae; *pa*, paraantennal setae; *preant*., preantennal setae; *ment.*, mental setae; *pm*, paramental setae; *max. palp.*, maxillary palpi; *max.*, maxillary setae; *pal. scl.*, pallete sclerite; *lun. n.*, lunar nodi; *tent. n.*, tentorial nodi; *ant. n.*, antennal nodi. (B) Thorax, ventrodorsal view: *L*, lateral prothoracic setae; *pr*, dorsal prothoracic setae; *prst. pl.*, prosternal plate; *prst. s.*, prosternal plate setae; *q*, setae ventrally and submarginally on the pterothorax; *st. s.*, sternal setae; *c*, four pairs of setae dorsally on the pterothorax; *w*, series of lateral setae on the pterothorax; *b*, dorsal setae on the posterior margin. (C) Abdomen terminus. *term. s.*, terminal setae of tergite IX; *vps*, ventral pleural setae; *I*, long pilose seta; *i*, short pilose seta; *S*, large spine; *s*, small spine (spines are on pleurites II–IV); *ADF*, dorsal anal fringe; *AVF*, ventral anal fringe. (D) Mandibles and ornamented ovoid sclerite of the nymph: *ov. scl.*, ovoid sclerite. (E) Abdomen, dorsoventral: *sen.*, sensilla; *spir.*, spiracle; *dps*, dorsal pleural setae; *ps.s.*, postspiracular setae; *VI–VIII*, tergites; *sc*, sternal central setae; *sl*, sternal lateral setae. Scale bar is 0.2 mm for all figures.

Type host: *Melanocorypha calandra* (L., 1766) – Calandra Lark (Alaudidae).

Type material: Holotype ♀ (slide no. MV21) and paratype ♀ with 1N (slide no. MV22) *ex M. calandra* (Passeriformes: Alaudidae), Calandra Lark. Azerbaijan. Kaspiy r. Viljash-Chay: 17–18.IV.1937, Ivanov coll. Collection of chewing lice of the Zoological Institute of the Russian Academy of Sciences, Saint Petersburg, Russia (ZISP) (Table 1).

**Table 1.** A list of chewing lice of the genus *Ricinus* deposited at the Zoological Institute of the Russian Academy of Sciences, Saint Petersburg, Russia.

Chewing lice	Host name	♀	♂	N	AZ	KY	RU	TJ	UA
*R. dolichocephalus* (Scopoli, 1763)	*Oriolus oriolus* (L., 1758)	1					+		
*R. elongatus* (Olfers, 1816)	*Turdus ruficollis Pallas, 1776	1					+**		
	*Turdus* sp.	5	1	1			+		
*R. frenatus* (Burmeister, 1838)	*Regulus regulus* (L., 1758)	7						+	
	*R. regulus buturlini* Loudon, 1911	3			+				
*R. fringillae* De Geer, 1778	*Carduelis flammea* (L., 1758) ***	1		4					
	*Emberiza aureola* Pallas, 1773	1		7			+**		
	*Emberiza citrinella* L., 1758	2	1					+	
	*Emberiza leucocephalos* Gmelin, 1771			1		+**			
	*Emberiza rustica* Pallas, 1776	2					+**		
	*Emberiza schoeniclus* (L., 1758)	1			+				
	*Fringilla coelebs* L., 1758	15	1	2		+**	+	+	
	*Passer montanus* (L., 1758)	3					+**		
	*Prunella collaris* (Scopoli, 1769)	1	1					+	
	*Prunella modularis* (L., 1758)	3					+**		
	*Turdus* sp.	1							+
*R. ivanovi* Blagoveshtchensky, 1951	*Leucosticte brandti* Bonaparte, 1851	1						+	
*R. rubeculae* (Schrank, 1776)	*Erithacus rubecula* (L., 1758)	2					+**		
	*Luscinia svecica* (L., 1758)	1					+**		
*R. serratus* (Durrant, 1906)	*Alauda arvensis* L., 1758	1					+**		
	*Calandrella acutirostris Hume, 1873	2						+**	
	*Galerida cristata* (L., 1758)	15	3	4				+	
*R. subdiffusus* Nelson, 1972^1^	*Emberiza* sp.	1					+		
*R. thoracicus* (Packard, 1870)	*Plectrophenax nivalis* (L., 1758)	1					+		
*R. tugarinovi* Blagoveshtchensky, 1951	*Terpsiphone paradisi* (L., 1758)	1						+	
*R. vaderi* n. sp.	*Melanocorypha calandra (L., 1766)	2		1	+**				
*Ricinus* sp. 1?	*Leucosticte brandti* Bonaparte, 1851			2		+**			
*Ricinus* sp. 2?	*Alauda arvensis* (L., 1758)	2					+		
*Ricinus* sp.^1^	*Buteo lagopus* (Pontoppidan, 1763)	1					+		
*Ricinus* sp.^1^	*Strix aluco harmsi* (Zarudny, 1911)	1					+		
Total		78	7	22					

♀ = female, ♂ = male, N = nymphs, AZ = Azerbaijan, KY = Kyrgyzstan, RU = Russia, TJ = Tajikistan, UA = Ukraine, “+” if collected in titled country

* host-louse records not cited by Price et al., 2003

** new country record

*** no locality notes

? specimens differ from other specimens from the same host species

1 stragglers.

Etymology: This species name is derived from Darth Vader, a fictional character in the Star Wars trilogy. The first author’s fiancée noticed a similarity between the head of the *R. vaderi* and Darth Vader’s helmet.

Authorship: Note that the authors of the new taxon are different from the authors of this paper; Article 50.1 and Recommendation 50A of the International Code of Zoological Nomenclature [12].

Description: Female (*n* = 2). Head wider than longer, with characteristic shape as (Fig. 2A); frons convex with rounded lateral margins, bearing dorsally pair of *df* setae and marginally 10 *f* setae; margin not continuous with that of marginal carinae. Lateral margin broadly concave. Temples expanded, outer margin curves inwardly, ending without hook-like structure; *t3* one half the size of *t1* and *t2*. Setae *a1* short, each associated with two sensillae; *a2* positioned marginal; *a3* absent. Lunar nodi present, twice the size of tentorial nodi. Setae along antennal lappets reduced and create diastoma, numbers vary in range 6–9 with 3–4 setae associated with *po* setae. Preantennal setae spinose. Setae *m4* same size as *pa* setae. Mandibles as in Figure 2D, monomorphic without finger-like extension. Ovoid sclerite round, compact with deeply pitted ornamentation. Labrum with setal pattern as in Figure 2A, pair 4 positioned slightly anteriorly to other setae in posterior row. Labium with 13 setae and pattern as in Figure 2A. Mental setae longer than maxillary setae; maxillary palpi genticuloid, extending past edge of head; maxillary plate sausage-shaped, relatively narrow. Gular plate as in Figure 2A with extensions reflexed outwardly; bearing two setae on each side.

Thorax (Fig. 2B). Setae *L3* and *L4* slightly pilose, one *L6* seta and small seta *L9*. Prosternal plate without nodi, pear-shaped; prosternal setae narrowly separated (0.04 mm). Two tactile setae on coxa I. *q2–q4* large and spinose, *q4* sometimes absent; *w* series composed of four setae with anterior two spinose and twice as large as posterior two pilose setae; *c1* spinose and larger than *c2*; *c2–c4* pilose. Sternal plate as in Figure 2B bearing one moderately long posterior seta and two or three short anterior setae.

Terminal segments of abdomen as in Figures 2C and 2E. Chaetotaxy in segments II through VII follow *Ricinus* pattern, two setae with inner less than half the length of the outer. One pair of long setae on sternite VIII and one vulval seta. Setae in anal fringe: ADF, 41–42; AVF, 42–43. Ventral pleural chaetotaxy as in Figures 2C and 2E: II, SSS; III, sss; IV, SIS; V, iIi; VI, iIi; VII, iIi and VIII, iIi. Terminal setae as in Figure 2C with small outer pair of setae, followed by two long setae, and two pairs of small inner setae. Pleural nodi unique (Fig. 2E).

Dimensions as follows: total length = 3.73–3.82; total width = 1.11; head length = 0.72–0.73; head width = 0.83; HI = 86–87 (head index = ratio of head length to head width × 100); labrum width = 0.36–0.37; prothorax length = 0.39–0.40; prothorax width = 0.73–0.75; distance between prosternal setae = 0.039–0.040.

Diagnosis: Our specimens of *R. vaderi* have ornamented ovoid sclerites, present only in the *diffusus*, *serratus* and *subangulatus* species groups. *Ricinus vaderi* cannot be placed into any of these groups. In the *subangulatus* species group, the frontal margin is continuous with that of marginal carinae, but not in *R. vaderi* n. sp. or the *diffusus* and *serratus* species groups. *Ricinus vaderi* n. sp. has a characteristic head shape with broadly concave lateral head margins; the head is wider than the length (HI = 86–87); setae along the antennal lappets are reduced with diastoma; the setal pattern on the terminal tergite iiIIi × iIIii. In the *diffusus* species group, the lateral margin is almost straight; HI is 100–110, with the exception of *R. thoracicus* (HI = 93); there is no reduction or diastoma along the antennal lappets and the pattern on terminal tergite iIIim × miIIi (where “m” means moderately long seta). *Ricinus serratus*, the only member of its group, has serrated pleural nodi, the head has a postfrontal constriction, the gular plate has no posterior extensions, the *a1* setae are long and there is one tactile seta on coxa I.

Remarks: *Ricinus vaderi* n. sp. is the second *Ricinus* species found on larks (Alaudidae).

### *Ricinus* sp. 1

Material examined: 2♀ (slide no. MV23) *ex Alauda arvensis* (L., 1758) (Passeriformes: Alaudidae), Sky Lark. Russia. 1904, Haritonov coll.

Remarks: The condition of these specimens is poor and we were not able to identify them. These two specimens differ from *R. serratus*, the only known *Ricinus* from this host species, in not having a laterally positioned, serrated structure on the pleurites.

### *Ricinus* sp. 2

Material examined: 2N (slide no. MV24) *ex Leucosticte brandti* (Passeriformes: Fringillidae), Black-headed Mountain-Finch. Kyrgyzstan. Naryn: 3.III.1913.

Remarks: These specimens are not identified to species level because of their poor condition, but they are not *R. ivanovi*, a known *Ricinus* from this host species. In contrast to *R. ivanovi*, the heads of these nymphs are wider than the length and have broadly concave lateral margins.

### *Ricinus* spp. Additional samples

Material examined: 1♀ (slide no. MV25) *ex Buteo lagopus* (Pontoppidan, 1763) (Falconiformes, Accipitridae), Rough-legged Hawk. Russia. Okhotsk: 25.V.1936

1♀ (slide no. MV26) *ex Strix aluco harmsi* (Zarudny, 1911) (Strigiformes, Strigidae), Tawny Owl (Turkestan). Russia. 17.V.1947, Ivanov coll.

Remarks: Ricinidae are parasites of songbirds (Passeriformes) and hummingbirds (Apodiformes: Trochilidae); hence, *Ricinus* spp. 3 and 4, whose specimens differ considerably from each other probably represent two different species. They are considered as contaminants (stragglers).

## Conclusions

The collection of chewing lice at the Zoological Institute of the Russian Academy of Sciences, Saint Petersburg, Russia was mainly assembled by Blagoveshtchensky in the 1930s through the 1970s. He published several surveys on the biodiversity of chewing lice in the former USSR [2–4]. We examined some specimens of *Ricinus* among his records. Exceptions are specimens of *R. fringillae ex E. cia* reported in Tajikistan [3], which have not been found in this collection. From Azerbaijan, we have recorded *Ricinus vaderi* n. sp. *ex Melanocorypha calandra*, and include previous records [2], bringing the total number of known *Ricinus* species in this country to three. We found only a single paper about chewing lice from Kyrgyzstan published by Kravtsova [13], who did not record any *Ricinus*. Our records represent the first findings of this genus in Kyrgyzstan. In addition to records of *Ricinus thoracicus* Packard, 1870 on Wrangel Island [4] and of *Ricinus elongatus* from two hosts and *R. fringillae* [14] in Central Ciscaucasia (northern part of the Caucasus region between the Black Sea and the Caspian Sea and within European Russia), herein we provide 10 new host-louse records in Russia. The presence of *R. elongatu*s *ex Turdus ruficollis*, *R. ivanovi ex Leucosticte tephrocotis*, *R. serratus* on *Calandrella acutirostris* and *C. cheleensis* was not cited in Price et al.’s world checklist [19]. We also designated lectotype specimens for *R. tugarinovi* and *R. ivanovi*.

We consider the genus *Ricinus* in a restricted sense, parasitizing only Passeriformes despite the recent study by Rheinwald [21] who synonymized *Trochiliphagus* with *Ricinus*. In his revision of the genus *Trochiliphagus*, he declared that all 12 known species of the genus *Trochiliphagus* should be one single species *Ricinus jimenezi*.

Rheinwald based his study on investigation of only two *Trochiliphagus* specimens from the same host, and did not examine any of the type material. This leaves some open questions: 1. Is there indeed only one species of *Trochiliphagus/Ricinus* parasitizing hummingbirds or more, regardless of the correct genus? 2. Is the status of the genus *Trochiliphagus* valid or is it indeed part of the genus *Ricinus*? 3. Perhaps the genus *Ricinus* occurring on Passeriformes could also be divided into several genera since some species groups of this genus (*dolichocephalus* species group) are more similar to those of *Trochiliphagus* from hummingbirds than to other species groups from passerine birds (for example, *arcuatus* group). As Rheinwald noted, application of modern enzymatic and DNA techniques may provide the answers. Therefore, until proper DNA studies are performed or at least morphological investigations of more specimens from different hummingbird species and of course of type material for the genus *Trochiliphagus*, we consider Rheinwald’s hypothesis is insufficiently supported and suggest resurrection of the name *Trochiliphagus*.
